# Rapid Detection of Hypervirulent Serovar 4h *Listeria monocytogenes* by Multiplex PCR

**DOI:** 10.3389/fmicb.2020.01309

**Published:** 2020-06-26

**Authors:** Youwei Feng, Hao Yao, Sisi Chen, Xiaowen Sun, Yuelan Yin, Xin’an Jiao

**Affiliations:** ^1^Jiangsu Key Laboratory of Zoonosis, Yangzhou University, Yangzhou, China; ^2^Key Laboratory of Prevention and Control of Biological Hazard Factors (Animal Origin) for Agrifood Safety and Quality, MOA of China, Yangzhou University, Yangzhou, China; ^3^Joint International Research Laboratory of Agriculture and Agri-Product Safety, Jiangsu Co-Innovation Center for Prevention and Control of Important Animal Infectious Disease and Zoonosis, Yangzhou University, Yangzhou, China

**Keywords:** *Listeria monocytogenes*, serovar 4h, multiplex PCR, identification, hypervirulent, somatic antigen, *Listeria ivanovii*

## Abstract

*Listeria monocytogenes* (*L. monocytogenes*) is a ubiquitous foodborne pathogen that comprises 14 serotypes, of which serovar 4h is a novel serotype recently reported. Serovar 4h *L. monocytogenes* belonging to hybrid sub-lineage II exhibit hypervirulent features. Conventional biochemical tests and widely used PCR-based serogrouping schemes could not distinguish serovar 4h strains. In this study, we developed a new multiplex PCR assay for rapid detection of serotype 4h *L. monocytogenes*. Three primer pairs based on the target genes, LMxysn_1095, *lmo1083*, and *smcL*, were designed. The multiplex PCR results showed that serovar 4h strains could be specifically identified from all tested strains, including various *L. monocytogenes* serovars, *Listeria* spp., and other species. The detection limits of the multiplex PCR were 291 fg/μL for genomic DNA and 5.5 × 10^6^ CFU/mL for bacterial suspension. Furthermore, pork meat artificially contaminated with serovar 4h *L. monocytogenes* in a concentration of 1.8 × 10^3^–1.8 × 10^0^ CFU/10 g were successfully detected within 10–16 h. These results demonstrate that the multiplex PCR with high specificity and sensitivity is applicable for the rapid detection of *L. monocytogenes* serotype 4h strains.

## Introduction

*Listeria monocytogenes* (*L. monocytogenes*) is a Gram-positive, facultative intracellular foodborne pathogen that is widely distributed in the environment (Vazquez-Boland et al., 2001; Liu et al., 2005). The bacterium is capable of causing spontaneous abortion, sepsis, and meningoencephalitis with a mortality rate of 20–30% in human (Farber and Peterkin, 1991; Barbuddhe and Chakraborty, 2009). Immunocompromised patients, pregnant women, newborns, and the elderly are at a greater risk of listeriosis (Lomonaco et al., 2009; Radoshevich and Cossart, 2017). Although the minimum infection dose of *L. monocytogenes* is at least 100 colony forming units (CFU) per gram of food (Yang et al., 2007), considering the possible serious consequences, many countries, including the United States, Australia, and China, have issued a zero-tolerance policy for *L. monocytogenes* in ready-to-eat (RTE) food (Churchill et al., 2006; Tao et al., 2016).

Because of the genetically heterogeneous nature of *L. monocytogenes*, serotyping as the primary characterization in epidemiological surveillance has been widely accepted. Based on somatic (O) and flagellar (H) antigens, 13 serotypes of *L. monocytogenes* have been described and divided into four phylogenetic subdivisions, lineages I to IV (Seeliger and Höhne, 1979; Ward et al., 2008). Lineage I includes serotypes 1/2b, 3b, 4b, 4d, 4e, and 7; lineage II includes serotypes 1/2a, 1/2c, 3a, and 3c; lineage III and IV include serotypes 4a, 4ab, 4c, and atypical 4b (Roberts et al., 2006; Ward et al., 2008; Orsi et al., 2011). In recent years, many PCR-based methods have been developed and widely used for *L. monocytogenes* serotyping. Doumith et al. (2004) established a PCR-serogroup method to subdivide *L. monocytogenes* into four molecular groups: 1/2a-3a, 1/2b-3b-7, 1/2c-3c, and 4b-4d-4e. Chen and Knabel (2007) proposed a multiplex PCR, which could detect *Listeria* spp. *L. monocytogenes* serotypes 1/2a and 4b and epidemic clones I, II, and III simultaneously. Nho et al. (2015) distinguished *L. monocytogenes* high-risk serotypes 1/2a and 1/2c in lineage I from low-risk serotypes 3a and 3c using a multiplex PCR assay. Alía et al. (2019) developed a quadruplex real-time quantitative PCR (qPCR) method capable of differentiating the four most predominant *L. monocytogenes* serotypes (1/2a, 1/2b, 1/2c, and 4b).

Serovar 4h was reported by Yin et al. (2019), that is, the 14th serotype of *L. monocytogenes* belonging to a hybrid sublineage of the major lineage II (HSL-II). *L. monocytogenes* serovar 4h strains are atypical because they fail to ferment rhamnose and cannot be recognized by the commonly accepted PCR serotyping method of Doumith et al. (2004). Strikingly, *L. monocytogenes* serotype 4h isolates harbor pan-species virulence genes acquiring from truncated pathogenicity island 2 (LIPI-2) of *Listeria ivanovii* (*L. ivanovii*) and gene cluster associated with galactosylation of wall teichoic acid (WTA) from Lineage I serovar 4b strains, which leads to hypervirulent properties of the bacteria (Yin et al., 2019). Therefore, it is urgent to implement robust monitoring and develop a rapid method of detecting *L. monocytogenes* serotype 4h strains.

In this study, we establish a multiplex PCR to distinguish *L. monocytogenes* serotype 4h from other serogroups and evaluate the specificity, sensitivity, and stability of this method. Besides, natural-occurring *L. monocytogenes* isolates and artificially contaminated meat samples were tested by the PCR assay. The multiplex PCR will be a useful tool for detection of serotype 4h *L. monocytogenes* from different sources of samples.

## Materials and Methods

### Bacterial Strains and Culture Conditions

The strains used in this study were commercially available or isolated by our laboratory, including 143 strains of *Listeria* spp. and 14 strains of other species. All tested strains were grown in brain heart infusion (BHI) (BD) or Luria-Bertani medium (LB) (OXOID), respectively, at 37°C overnight under constant shaking.

### Target Genes

To differentiate serotype 4h *L. monocytogenes* from other *L. monocytogenes* serogroups by multiplex PCR, we downloaded various *L. monocytogenes* serotypes and non-*L. monocytogenes* genome sequences from the GenBank database^1^. Three target genes, LMxysn_1095 (GenBank accession no. CP007583.1 segment 1113983-1115773), *lmo1083* (Gene ID: 986257), and *smcL* (GenBank accession no. CP007583.1 segment 1245812-1246819), were selected by comparative genomics analysis. The corresponding primers were designed based on the conserved region of target genes by Primer Premier 5.0 software (PREMIER Biosoft International, Palo Alto, CA, United States) and determined by NCBI Primer-BLAST tool (Table 1).

**TABLE 1:** Nucleotide sequence of the primers used in this study.

Target gene	Primer sequence (5′-3′)	Product size	Protein encoded	Serotype specificity
LMxysn_1095	F: AATACTTGGACAGACGGAACGC	602 bp	Galactosyltransferase	Serotypes 4h, 4b, 4d, and 4e
	R: TCATCTGGCTCTTTTAGAACCG			
*lmo1083*	F: CACAAATGGTCTTGACGGGG	390 bp	L-rhamnosyl transferase	Serotypes 1/2, 3, and 7 and *Listeria seeligery* (1/2b)
	R: TTTGCGCGTGATTTTAGTGG			
*smcL*	F: CACAGACCATTGTGGTGACTTG	889 bp	Sphingomyelinase C	Serotype 4h and *L. ivanovii*
	R: CGGTGCTTTCATTTTTTTACTC			

*F, forward primer; R, reverse primer.*

### Genomic DNA Extraction

The extraction of *Listeria* genomic DNA was conducted by an optimized boiling lysis method. Bacteria culture (0.5–1 × 10^9^ CFU) was rinsed with phosphate-buffered saline and then centrifuged at 8000 rpm for 2 min. The pellet was mixed thoroughly with 88 μL of sterile distilled water and 10 μL of lysozyme (20 mg/mL) and incubated at 37°C for 10 min; after that, 1 μL of protease K (20 mg/mL) and 1 μL of RNase A (10 mg/mL) were added and incubated at 58°C for 10 min. Finally, the suspension was heated at 95°C for 5 min and centrifuged at 12,000 rpm for 2 min. The supernatant was used as the DNA template for multiplex PCR. The concentration of genomic DNA was measured by a NanoDrop 2000 spectrophotometer (Thermo Fisher Scientific, United States).

### Multiplex PCR

The multiplex PCR mixture contained 12.5 μL of 2× Taq Master mix (Vazyme Biotech Co., Ltd., China), 0.4 μM of each primer, bacterial genomic DNA, and ultrapure water to fill the final volume to 25 μL. The PCR procedure consisted of an initial denaturation step at 95°C for 5 min; 30 cycles of quantification at 95°C for 0.5 min, 55°C for 0.5 min, and 72°C for 1 min, and a final cycle at 72°C for 10 min. The amplicons were separated on 1% agarose gels (ethidium bromide dyed) in 1× TAE buffer and visualized using a Gel Doc XR system (Bio-Rad, Hercules, CA, United States).

### Specificity Evaluation of Multiplex PCR

The specificity of multiplex PCR for detection of serovar 4h *L. monocytogenes* was evaluated. The genomic DNA was extracted from 14 strains of *L. monocytogenes* (including three serovar 4h strains) and 14 strains of other species (Table 2), and 1 μL of genomic DNA was used as the PCR template.

**TABLE 2:** *Listeria* spp. and other strains used in this study for multiplex PCR specificity evaluation.

Species (number of isolates)	Strain	Serotype	PCR results (LMxysn_1095/*lmo1083*/*smcL*)
*L. monocytogenes* (14)	XYSN	4h	+/−/+
*L. monocytogenes* (14)	15LG	4h	+/−/+
*L. monocytogenes* (14)	16E	4h	+/−/+
*L. monocytogenes* (14)	EGD-e	1/2a	−/+/−
*L. monocytogenes* (14)	LmBJ113	1/2b	−/+/−
*L. monocytogenes* (14)	LmBJ114	1/2c	−/+/−
*L. monocytogenes* (14)	LmBJ115	3a	−/+/−
*L. monocytogenes* (14)	LmBJ117	3c	−/+/−
*L. monocytogenes* (14)	LmBJ118	4a	−/−/−
*L. monocytogenes* (14)	LmBJ119	4ab	−/−/−
*L. monocytogenes* (14)	NTSN	4b	+/−/−
*L. monocytogenes* (14)	LmBJ121	4d	+/−/−
*L. monocytogenes* (14)	LmBJ122	4c	−/−/−
*L. monocytogenes* (14)	LmBJ123	7	−/+/−
*L. ivanovii* (2)	YZU0805	–	−/−/+
	LIV ZJU	–	−/−/+
*Listeria innocua* (1)	LBJ131	–	−/−/−
*Listeria grayi* (1)	LBJ132	–	−/−/−
*Listeria seeligery* (1)	LBJ133	–	−/+/−
*Listeria welshimeri* (1)	LBJ137	–	−/−/−
*S.* Enteritidis (1)	C50041	–	−/−/−
*Salmonella* Pullorum (1)	S06004	–	−/−/−
*Salmonella* Typhimurium (1)	–	–	−/−/−
*Vibrio parahaemolyticus* (1)	–	–	−/−/−
*E. coli* (1)	ATCC25922	–	−/−/−
*Staphylococcus aureus* (1)	–	–	−/−/−
*Campylobacter jejuni* (1)	–	–	−/−/−
*Campylobacter coli* (1)	–	–	−/−/−

### Detection Limits Evaluation of Multiplex PCR

For detection limit evaluation, genomic DNA of serovar 4h *L. monocytogenes* strain XYSN was extracted and serially diluted 10-fold from 291 ng/μL to 291 ag/μL. Meanwhile, bacteria suspension of the strain XYSN was serially diluted 10-fold from 5.5 × 10^9^ to 5.5 × 10^0^ CFU/mL, and 1 μL of genomic DNA and 5 μL of bacteria suspension as the PCR template were added to the PCR mixture, respectively. The detection limits of the multiplex PCR were judged by electrophoresis.

### Stability Evaluation of Multiplex PCR

Stability evaluation of multiplex PCR was conducted, referring to the methodology by Tao et al. (2016). Simply, serotype 4h *L. monocytogenes* strain XYSN, *Escherichia coli* (*E. coli*) strain ATCC25922, and *Salmonella* Enteritidis (*S.* Enteritidis) strain C50041 were cultured at 37°C overnight and serially 10-fold diluted. The concentration of serotype 4h *L. monocytogenes* was set at 10^4^ CFU/mL and the other two strains were prepared from 10^4^ to 10^9^ CFU/mL. The bacteria culture of serotype 4h *L. monocytogenes* was mixed with *E. coli* and *S.* Enteritidis in ratios of 1:1, 1:10, 1:10^3^, and 1:10^5^. Genomic DNA of each mixture was extracted, and 3 μL of genomic DNA were used as the template for multiplex PCR.

### *Listeria monocytogenes* Isolates Testing

Naturally contaminated samples between September 2018 to September 2019 were collected from goat farms, slaughterhouses, and markets in Jiangsu, China, according to the testing methodology for *Listeria* species or *L. monocytogenes* in environmental samples (Anonymous, 2015). Briefly, all samples were incubated in UVM broth (BD) at 30°C for 24 h. Then, the UVM enrichment (100 μL) was transferred to Fraser broth (BD) at 37°C for 24 h. Afterward, the culture was streaked onto CHROMagar^TM^ plates and incubated at 37°C for 36 h. Colonies with a blue/green sheen and opaque halos were purified on BHI agar plates and confirmed by VITEK-2 system (bioMérieux, France). To evaluate the efficacy of the multiplex PCR for identifying serotype 4h strains, all *L. monocytogenes* isolates were further subjected to the developed multiplex PCR assay. Meanwhile, the serovar of isolates was determined by traditional slide agglutination test (Denka-Seiken Co., Ltd., Japan). The results obtained by multiplex PCR and the agglutination test were compared according to ISO 16140:2003 (Anonymous, 2003).

### Artificial Contamination Experiment

The artificial contamination experiment was performed based on the procedure by Sheng et al. (2018). Briefly, *L. monocytogenes* serotype 4h strain XYSN was cultured overnight at 37°C and then prepared at different concentrations ranging from 10^1^ to 10^5^ CFU/mL. The raw pork was fully sterilized by UV for half an hour in advance. Each dilution of bacteria (100 μL) was sprayed separately on the upper surface of 10 g meat (around 10 cm^2^). When the bacteria suspension on the surface was completely dry, the artificially contaminated pork was put into sampling bags containing 90 mL BHI medium and incubated at 37°C for 4, 6, 8, 10, and 12 h. Genomic DNA were extracted from 1 mL of BHI medium and detected by multiplex PCR. The experiment was repeated three times.

## Results

### Designing of Primers

LMxysn_1095 and *lmo1083* are somatic antigen-related genes involved in glycosylation modification of the WTA in *L. monocytogenes* (Carvalho et al., 2015; Yin et al., 2019). Comparative genomics analysis revealed that LMxysn_1095 presents in *L. monocytogenes* serovars 4h, 4b, 4d, and 4e, and *lmo1083* presents in serovars 1/2, 3, and 7 as well as *Listeria seeligery* (*L. seeligery*, 1/2b). *smcL* is one of the virulence factors located on the LIPI-2 of *L. ivanovii* (Rodríguez-Lázaro et al., 2010; Wang et al., 2014). Genome of serotype 4h *L. monocytogenes* carry a partial sequence from LIPI-2 of *L. ivanovii*, which contains the *smcL* gene. Therefore, three primer pairs were designed to distinguish *L. monocytogenes* serotype 4h from other serovars, specifically targeting LMxysn_1095, *lmo1083*, and *smcL* (Table 1).

### Specificity of Multiplex PCR

Twenty-eight bacteria strains were used to detect the specificity of multiplex PCR. The results showed that strains of *L. monocytogenes* serotype 4h generated two amplicons of 602 and 889 bp for the LMxysn_1095 and *smcL*, respectively. Strains of *L. monocytogenes* serovars 4b and 4d amplified the 602 bp band for LMxysn_1095. *L. ivanovii* generated the 889 bp band for *smcL.* Strains of *L. monocytogenes* serovars 1/2a, 1/2b, 1/2c, 3a, 3c, and 7 and *L. seeligery* generated the 390 bp band for *lmo1083*. The remaining strains presented no amplicon (Table 2). It was concluded that the multiplex PCR can specifically differentiate serovar 4h *L. monocytogenes* from other strains through the characteristic PCR band patterns.

### Detection Limits of Multiplex PCR

**FIGURE 1:**
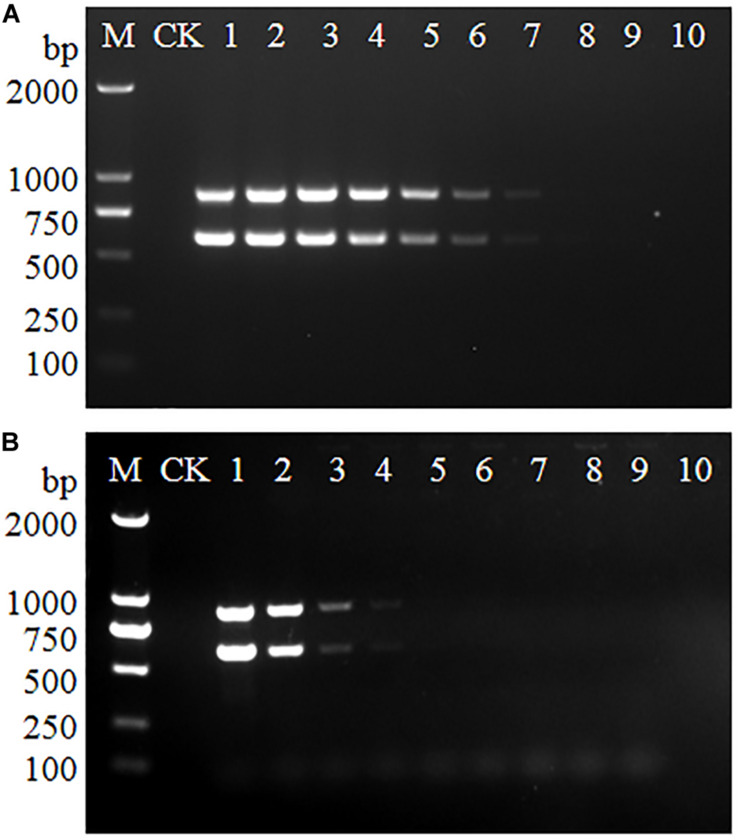
Detection limits of multiplex PCR for *L. monocytogenes* serotype 4h. The genomic DNA **(A)** and bacteria suspension **(B)** of serotype 4h *L. monocytogenes* were used as the PCR templates. Lane M: DL2000 DNA ladder; Lane CK: negative control. **(A)** Lanes 1–10: 291 ng/μL, 29.1 ng/μL, 2.91 ng/μL, 291 pg/μL, 29.1 pg/μL, 2.91 pg/μL, 291 fg/μL, 29.1 fg/μL, 2.91 fg/μL, 291 ag/μL. **(B)** Lanes 1–10: 5.5 × 10^9^, 5.5 × 10^8^, 5.5 × 10^7^, 5.5 × 10^6^, 5.5 × 10^5^, 5.5 × 10^4^, 5.5 × 10^3^, 5.5 × 10^2^, 5.5 × 10^1^, 5.5 × 10^0^ CFU/mL.

To determine the detection limits of the multiplex PCR, serially diluted genomic DNA and bacteria suspension of serotype 4h *L. monocytogenes* were used as the PCR template. As shown in Figures 1A,B, the 602 and 889 bp bands could be observed when the genomic DNA concentration ranged from 291 ng/μL to 291 fg/μL and the bacteria suspension concentration decreased from 5.5 × 10^9^ to 5.5 × 10^6^ CFU/mL. The results indicate that at least 291 fg/μL of genomic DNA or 5.5 × 10^6^ CFU/mL of bacteria suspension are needed for multiplex PCR detection of serovar 4h *L. monocytogenes*.

### Stability of Multiplex PCR

**FIGURE 2:**
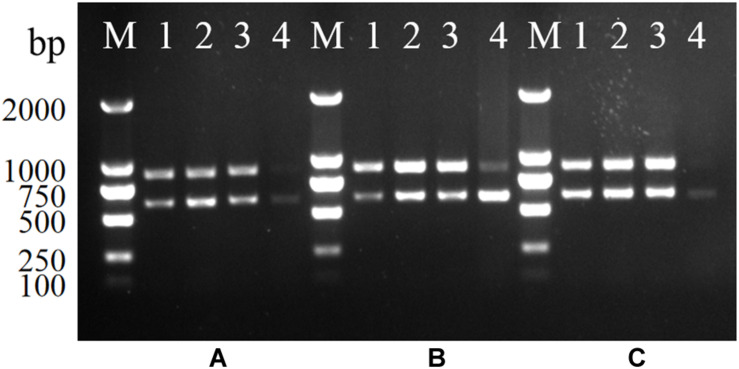
Stability of the multiplex PCR for *L. monocytogenes* serotype 4h. **(A–C)**: serotype 4h *L. monocytogenes* were mixed with *S*. Enteritidis **(A)**, *E. coli* **(B)**, and two bacteria co-cultures **(C)**. Lanes 1–4: bacteria suspension of serotype 4h mixing with other bacteria in ratios of 1:1, 1:10, 1:10^3^, and 1:10^5^; Lane M: DL2000 DNA ladder.

Serovar 4h *L. monocytogenes* was mixed with *E. coli*, *S*. Enteritidis, and two bacteria co-cultures at the ratios of 1:1, 1:10, 1:10^3^, and 10^5^. The genomic DNA of each mixture was extracted and used as PCR templates. As shown in Figure 2, two specific bands were generated for each mixture, indicating that the multiplex PCR was not affected by other interference strains present in a multicomponent sample.

### Identification of *Listeria monocytogenes* Isolates

All 129 *L. monocytogenes* isolates from food and environmental samples were identified by multiplex PCR and serotyped by slide agglutination in parallel (Table 3 and Supplementary Table S1). A traditional slide agglutination test showed that the 129 isolates belonged to six serovars (1/2a, 1/2b, 1/2c, 3c, 4b, and 4h), including two serotype 4h strains. The PCR results suggest that two isolates generated two specific bands for LMxysn_1095 and *smcL*, which were identified as serotype 4h *L. monocytogenes*. These results were consistent with that of the slide agglutination test. All serovar 4h isolates were identified using the multiplex PCR method, and no amplification was found for non-serovar 4h strains, indicating that the relative sensitivity and specificity of the multiplex PCR were both 100%.

**TABLE 3:** *L. monocytogenes* serotype 4 strains isolated from diverse sources.

Isolate no.	Serotype	PCR results (LMxysn_1095/*lmo1083*/*smcL*)	Sample types	Sources
P161020-B18	4h	+/−/+	Environment	Goat farm
P161020-D14	4h	+/−/+	Environment	Goat farm
Lm03032001	4b	+/−/−	Raw pork meat	Market
Lm03061801	4b	+/−/−	Raw pork meat	Market
Lm03060104	4b	+/−/−	Environment	Market
Lm03061102	4b	+/−/−	Environment	Market
Lm03061104	4b	+/−/−	RTE meat	Market
Lm03061807	4b	+/−/−	RTE meat	Market
Lm03061808	4b	+/−/−	RTE meat	Market

### Detection of Artificially Contaminated Meat

The multiplex PCR was applied to detect serotype 4h *L. monocytogenes* in artificially contaminated pork. The initial bacterial inoculation dose per 10 g of meat was 1.8 × 10^4^, 1.8 × 10^3^, 1.8 × 10^2^, 1.8 × 10^1^, and 1.8 × 10^0^ CFU. Afterward, all samples were incubated at 37°C in BHI medium for 4, 6, 8, 10, and 12 h. As shown in Table 4 and Supplementary Figure S1, with the prolongation of enrichment time, the detection limit of the PCR assay gradually improved. After 6, 8, 10, and 12 h of enrichment, the detection limit of multiplex PCR was 1.8 × 10^3^, 1.8 × 10^2^, 1.8 × 10^1^, and 1.8 × 10^0^ CFU/10 g, respectively. Considering the time required for other operation procedures (genomic DNA extraction, PCR, and electrophoresis), it can be speculated that meat samples with the contamination dose less than 1.8 × 10^3^ CFU/10 g can be detected within 10–16 h.

**TABLE 4:** Detection of artificially contaminated pork by multiplex PCR.

Initial inoculum	Multiplex PCR results of				
(CFU/10 g)	different enrichment time				
	4 h	6 h	8 h	10 h	12 h
1.8 × 10^4^	−	+	+	+	+
1.8 × 10^3^	−	+	+	+	+
1.8 × 10^2^	−	−	+	+	+
1.8 × 10^1^	−	−	−	+	+
1.8 × 10^0^	−	−	−	−	+
0	−	−	−	−	−

*+, positive PCR result; −, negative PCR result.*

## Discussion

*Listeria monocytogenes* is the main etiology of the fatal disease listeriosis, which poses a serious threat to human health. The vast majority of listeriosis clinical cases were caused by *L. monocytogenes* serotypes 1/2a, 1/2b, and 4b (Vines and Swaminathan, 1998). Hypervirulent serotype 4h *L. monocytogenes* were discovered and reported from goat listeriosis outbreaks and environments. Newly named serotype 4h can be identified by slide agglutination that reacts positively to O- antigens V/VI and VI and to H-antigens A and B (Yin et al., 2019), but this method is limited by the high cost of antisera and only if it is the pure colony. Thus, establishment of a rapid detection method for serotype 4h *L. monocytogenes* is of great significance for effective prevention and control of the pathogen.

In this study, a multiplex PCR method comprising three primer pairs, LMxysn_1095, *lmo1083*, and *smcL*, was developed for detection of *L. monocytogenes* serotype 4h. The sensitivity of the multiplex PCR was evaluated using both serovar 4h *L. monocytogenes* pure culture and their genomic DNA. It was found that the detection limit of multiplex PCR using genomic DNA as the template is close to previously reported PCR methods for *L. monocytogenes* subtyping (Tao et al., 2016; Razei et al., 2017; Sheng et al., 2018). However, the detection limit from bacteria culture was lower than genomic DNA because of the impact of the cell wall, nuclease, and metabolites in bacterial suspension (Sheng et al., 2018). Low contamination levels in samples make it difficult to detect target pathogens; hence, enrichment steps were applied in the process of *L. monocytogenes* detection (Garrido et al., 2013; Ding et al., 2017; Parichehr et al., 2019). Target bacteria and background flora, such as *E. coli* and *Salmonella*, coexist in enrichment medium, which may interfere with the stability of detection methods. Nevertheless, the multiplex PCR was not affected by existence of other strains even though the genomic DNA concentration of background flora was significantly higher than the target bacteria.

Contamination of meat products with *L. monocytogenes* is a public health concern because many human listeriosis outbreaks have been linked to contaminated meat products (Jacquet et al., 1995; Loncarevic et al., 1997; Jensen et al., 2016; Anna et al., 2018). Thus, rapid detection of *L. monocytogenes* from contaminated meat, etc., provides an effective guarantee for the food safety system. Conventional detection methods for *L. monocytogenes* are laborious and time-consuming, including procedures of primary isolation with enrichment and selective medium, biochemical analysis and serological tests, which take up to 5–10 days (Grady et al., 2008; Vanegas et al., 2009; Frece et al., 2010). In the artificial contamination experiment of this study, the multiplex PCR could detect serotype 4h *L. monocytogenes* in meat with a contaminated concentration of less than 1.8 × 10^3^ CFU/10 g within 10–16 h, which dramatically shortened the detection time (10–16 h vs. 5–10 days). It is noteworthy that the optimized boiling lysis method was applied to extract genomic DNA directly from enrichment medium as the PCR template. The full process could be accomplished in 25–30 min, which is more proper for rapid extraction of genomic DNA from large-scale samples than the conventional extraction method by kit (25–30 min vs. 3 h).

In general, the multiplex PCR could detect serovar 4h *L. monocytogenes* in artificially contaminated meat rapidly and accurately, which is expected to be adopted for laboratory or clinical tests of naturally contaminated samples. Up to now, the collection of *L. monocytogenes* serovar 4h isolates was limited; five 4h strains have been isolated from goat listeriosis and environment. More *L. monocytogenes* serovar 4h strains are possible to be detected with the multiplex PCR assay established in this study.

## Conclusion

In conclusion, a multiplex PCR method comprising three primer pairs, LMxysn_1095, *lmo1083*, and *smcL*, was proposed in this study. PCR results demonstrate that the multiplex PCR with high specificity, sensitivity, and stability is applicable to detect *L. monocytogenes* serotype 4h rapidly and effectively. Our research affords a useful detection and identification technique to enhance surveillance for the emergence of hypervirulent serovar 4h *L. monocytogenes*.

## Data Availability Statement

The raw data supporting the conclusions of this article will be made available by the authors, without undue reservation, to any qualified researcher.

## Author Contributions

YY and XJ designed the experiments. YF, HY, SC, and XS collected all strains, performed the PCR assays, and analyzed the results. YY, XJ, and YF wrote the manuscript. All authors read and approved the final manuscript.

## Conflict of Interest

The authors declare that the research was conducted in the absence of any commercial or financial relationships that could be construed as a potential conflict of interest.

## Footnotes

1 http://www.ncbi.nlm.nih.gov/nuccore/

## Supplementary Material

FIGURE S1 Detection results of pork meat contaminated with serotype 4h *L. monocytogenes* by multiplex PCR. Lanes 1–6: the initial bacterial inoculation dose was 0, 1.8×10^0^, 1.8×10^1^, 1.8×10^2^, 1.8×10^3^, and 1.8×10^4^ CFU, respectively; Lane M: DL2000 DNA ladder.
