# Supplementary material for: Rapid Detection of Hypervirulent Serovar 4h Listeria monocytogenes by Multiplex PCR
Source: Front Microbiol. 2020 Jun 26;11:1309. doi: 10.3389/fmicb.2020.01309 (PMC7333235; doi:10.3389/fmicb.2020.01309)
Supplement: Supplementary file 1 [file Table_1.docx]

**Supplementary Table 1.**

***L. monocytogenes* serotypes non- 4 strains isolated from diverse sources.**

| **NO.** | **Isolate no.** | **Serotype** | **PCR Results (LMxysn_1095/ *lmo1083*/ *smcL*)** | **Sample Types** | **Sources** |
| --- | --- | --- | --- | --- | --- |
| 1 | YZP18050548 | 1/2c | －/＋/－ | raw pork meat | market |
| 2 | YZP18051957 | 1/2a | －/＋/－ | raw pork meat | market |
| 3 | YZP18051962 | 1/2a | －/＋/－ | raw pork meat | market |
| 4 | YZP18051913 | 1/2a | －/＋/－ | raw pork meat | market |
| 5 | YZP18051934 | 1/2a | －/＋/－ | raw pork meat | market |
| 6 | YZP18051942 | 1/2a | －/＋/－ | raw pork meat | market |
| 7 | YZP18051927 | 1/2b | －/＋/－ | raw pork meat | market |
| 8 | YZP18051953 | 1/2c | －/＋/－ | raw pork meat | market |
| 9 | YZP18051966 | 1/2a | －/＋/－ | raw pork meat | market |
| 10 | YZP18060171 | 1/2a | －/＋/－ | raw pork meat | market |
| 11 | YZP18060118 | 1/2c | －/＋/－ | raw pork meat | market |
| 12 | XYP18062228 | 1/2a | －/＋/－ | raw pork meat | market |
| 13 | XYP18062256 | 1/2c | －/＋/－ | raw pork meat | market |
| 14 | XYP18062298 | 3c | －/＋/－ | raw pork meat | market |
| 15 | YZP1806302 | 1/2c | －/＋/－ | raw pork meat | market |
| 16 | YZP1806303 | 1/2c | －/＋/－ | raw pork meat | market |
| 17 | YZP1806307 | 1/2a | －/＋/－ | raw pork meat | market |
| 18 | YZP18063010 | 1/2c | －/＋/－ | raw pork meat | market |
| 19 | YZP18063011 | 1/2c | －/＋/－ | raw pork meat | market |
| 20 | YZP18063012 | 1/2a | －/＋/－ | raw pork meat | market |
| 21 | YZP18063016 | 1/2c | －/＋/－ | raw pork meat | market |
| 22 | YZP1807151 | 1/2c | －/＋/－ | raw pork meat | market |
| 23 | YZP1807152 | 1/2a | －/＋/－ | raw pork meat | market |
| 24 | YZP1807154 | 1/2c | －/＋/－ | raw pork meat | market |
| 25 | YZP1807155 | 1/2a | －/＋/－ | raw pork meat | market |
| 26 | YZP1807158 | 1/2a | －/＋/－ | raw pork meat | market |
| 27 | YZP1807159 | 1/2a | －/＋/－ | raw pork meat | market |
| 28 | YZP18071516 | 1/2c | －/＋/－ | raw pork meat | market |
| 29 | YZP18072902 | 1/2c | －/＋/－ | raw pork meat | market |
| 30 | YZP18072908 | 1/2a | －/＋/－ | raw pork meat | market |
| 31 | YZP18072909 | 1/2a | －/＋/－ | raw pork meat | market |
| 32 | YZP18072915 | 1/2a | －/＋/－ | raw pork meat | market |
| 33 | YZ18090703 | 1/2a | －/＋/－ | raw pork meat | market |
| 34 | YZ18090704 | 1/2c | －/＋/－ | raw pork meat | market |
| 35 | YZ18090706 | 1/2c | －/＋/－ | raw pork meat | market |
| 36 | YZ18090709 | 1/2a | －/＋/－ | raw pork meat | market |
| 37 | YZ18091403 | 1/2a | －/＋/－ | raw pork meat | market |
| 38 | YZ18091404 | 1/2c | －/＋/－ | raw pork meat | market |
| 39 | YZ18091406 | 1/2c | －/＋/－ | raw pork meat | market |
| 40 | YZ18091409 | 1/2a | －/＋/－ | raw pork meat | market |
| 41 | YZP18091901 | 1/2c | －/＋/－ | raw pork meat | market |
| 42 | YZ18100102 | 1/2a | －/＋/－ | raw pork meat | market |
| 43 | YZ18103108 | 1/2c | －/＋/－ | raw pork meat | market |
| 44 | YZ18103120 | 1/2c | －/＋/－ | raw pork meat | market |
| 45 | YZ18120201 | 1/2a | －/＋/－ | raw pork meat | market |
| 46 | YZ18120202 | 1/2c | －/＋/－ | raw pork meat | market |
| 47 | YZ18120203 | 1/2a | －/＋/－ | raw pork meat | market |
| 48 | YZ18120204 | 1/2a | －/＋/－ | raw pork meat | market |
| 49 | YZ18120205 | 1/2c | －/＋/－ | raw pork meat | market |
| 50 | YZ18120207 | 1/2c | －/＋/－ | raw pork meat | market |
| 51 | YZ18120208 | 1/2c | －/＋/－ | raw pork meat | market |
| 52 | YZ18120211 | 1/2a | －/＋/－ | raw pork meat | market |
| 53 | YZ18120213 | 1/2a | －/＋/－ | raw pork meat | market |
| 54 | YZ18122202 | 1/2c | －/＋/－ | raw pork meat | market |
| 55 | YZ18122203 | 1/2c | －/＋/－ | raw pork meat | market |
| 56 | YZ18122208 | 1/2a | －/＋/－ | raw pork meat | market |
| 57 | YZ18122213 | 1/2a | －/＋/－ | raw pork meat | market |
| 58 | YZ18122214 | 1/2a | －/＋/－ | raw pork meat | market |
| 59 | YZ18122215 | 1/2c | －/＋/－ | raw pork meat | market |
| 60 | YZ18122705 | 1/2a | －/＋/－ | raw pork meat | market |
| 61 | YZ18122706 | 1/2a | －/＋/－ | raw pork meat | market |
| 62 | YZ18122707 | 1/2c | －/＋/－ | raw pork meat | market |
| 63 | YZ18122708 | 1/2a | －/＋/－ | raw pork meat | market |
| 64 | YZ18122709 | 1/2c | －/＋/－ | raw pork meat | market |
| 65 | YZ18122710 | 1/2c | －/＋/－ | raw pork meat | market |
| 66 | YZ18122711 | 1/2a | －/＋/－ | raw pork meat | market |
| 67 | YZ18122712 | 1/2c | －/＋/－ | raw pork meat | market |
| 68 | YZ18122716 | 1/2c | －/＋/－ | raw pork meat | market |
| 69 | YZ18122718 | 1/2a | －/＋/－ | raw pork meat | market |
| 70 | YZ18122719 | 1/2a | －/＋/－ | raw pork meat | market |
| 71 | YZ18122720 | 1/2c | －/＋/－ | raw pork meat | market |
| 72 | YZ18122723 | 1/2a | －/＋/－ | raw pork meat | market |
| 73 | YZ18122724 | 1/2a | －/＋/－ | raw pork meat | market |
| 74 | YZ18122725 | 1/2a | －/＋/－ | raw pork meat | market |
| 75 | YZ18122727 | 1/2c | －/＋/－ | raw pork meat | market |
| 76 | YZ18122728 | 1/2c | －/＋/－ | raw pork meat | market |
| 77 | YZ19010301 | 1/2a | －/＋/－ | raw pork meat | market |
| 78 | YZ19010302 | 1/2a | －/＋/－ | raw pork meat | market |
| 79 | YZ19010303 | 1/2c | －/＋/－ | raw pork meat | market |
| 80 | YZ19010307 | 1/2a | －/＋/－ | raw pork meat | market |
| 81 | YZ19010308 | 1/2a | －/＋/－ | raw pork meat | market |
| 82 | YZ19010309 | 1/2a | －/＋/－ | raw pork meat | market |
| 83 | YZ19022509 | 1/2a | －/＋/－ | raw pork meat | market |
| 84 | YZ19022521 | 1/2c | －/＋/－ | raw pork meat | market |
| 85 | YZ19031801 | 1/2a | －/＋/－ | raw pork meat | market |
| 86 | YZ19031802 | 1/2a | －/＋/－ | raw pork meat | market |
| 87 | YZ19031804 | 1/2a | －/＋/－ | raw pork meat | market |
| 88 | YZ19031807 | 1/2a | －/＋/－ | raw pork meat | market |
| 89 | YZ19031808 | 1/2a | －/＋/－ | raw pork meat | market |
| 90 | YZ19031809 | 1/2c | －/＋/－ | raw pork meat | market |
| 91 | YZ19042201 | 1/2c | －/＋/－ | raw pork meat | market |
| 92 | YZ19052805 | 1/2a | －/＋/－ | raw pork meat | market |
| 93 | YZ19052806 | 1/2c | －/＋/－ | raw pork meat | market |
| 94 | YZ19052808 | 1/2a | －/＋/－ | raw pork meat | market |
| 95 | YZ19052809 | 1/2a | －/＋/－ | raw pork meat | market |
| 96 | YZ19052810 | 1/2a | －/＋/－ | raw pork meat | market |
| 97 | YZ19052811 | 1/2a | －/＋/－ | raw pork meat | market |
| 98 | HAP18061975 | 1/2a | －/＋/－ | chilling | slaughterhouse |
| 99 | HAP18061951 | 1/2c | －/＋/－ | chilling | slaughterhouse |
| 100 | HAPG1818080308 | 1/2c | －/＋/－ | chilling | slaughterhouse |
| 101 | HAPG1818080311 | 1/2a | －/＋/－ | chilling | slaughterhouse |
| 102 | HAPG1818080313 | 1/2c | －/＋/－ | chilling | slaughterhouse |
| 103 | HAPG1818080315 | 1/2c | －/＋/－ | chilling | slaughterhouse |
| 104 | HAPT1818080301 | 1/2c | －/＋/－ | transport | slaughterhouse |
| 105 | HAPT1818080302 | 1/2a | －/＋/－ | transport | slaughterhouse |
| 106 | HAPT1818080303 | 1/2a | －/＋/－ | transport | slaughterhouse |
| 107 | HA18090715 | 1/2a | －/＋/－ | chilling | slaughterhouse |
| 108 | HA18122609 | 1/2a | －/＋/－ | chilling | slaughterhouse |
| 109 | HA18122612 | 1/2a | －/＋/－ | chilling | slaughterhouse |
| 110 | HA18122614 | 1/2a | －/＋/－ | chilling | slaughterhouse |
| 111 | HA18122620 | 1/2a | －/＋/－ | chilling | slaughterhouse |
| 112 | HA18122713 | 1/2a | －/＋/－ | transport | slaughterhouse |
| 113 | HA18122715 | 1/2c | －/＋/－ | transport | slaughterhouse |
| 114 | HA18122716 | 1/2a | －/＋/－ | transport | slaughterhouse |
| 115 | HA18122718 | 1/2c | －/＋/－ | transport | slaughterhouse |
| 116 | HA18122720 | 1/2c | －/＋/－ | transport | slaughterhouse |
| 117 | HA19060802 | 1/2a | －/＋/－ | chilling | slaughterhouse |
| 118 | HA19060904 | 1/2a | －/＋/－ | transport | slaughterhouse |
| 119 | HA19060906 | 1/2a | －/＋/－ | transport | slaughterhouse |
| 120 | HA19060913 | 1/2a | －/＋/－ | transport | slaughterhouse |
