# Supplementary figures and images for: Rapid Detection of Hypervirulent Serovar 4h Listeria monocytogenes by Multiplex PCR
Source: Front Microbiol. 2020 Jun 26;11:1309. doi: 10.3389/fmicb.2020.01309 (PMC7333235; doi:10.3389/fmicb.2020.01309)

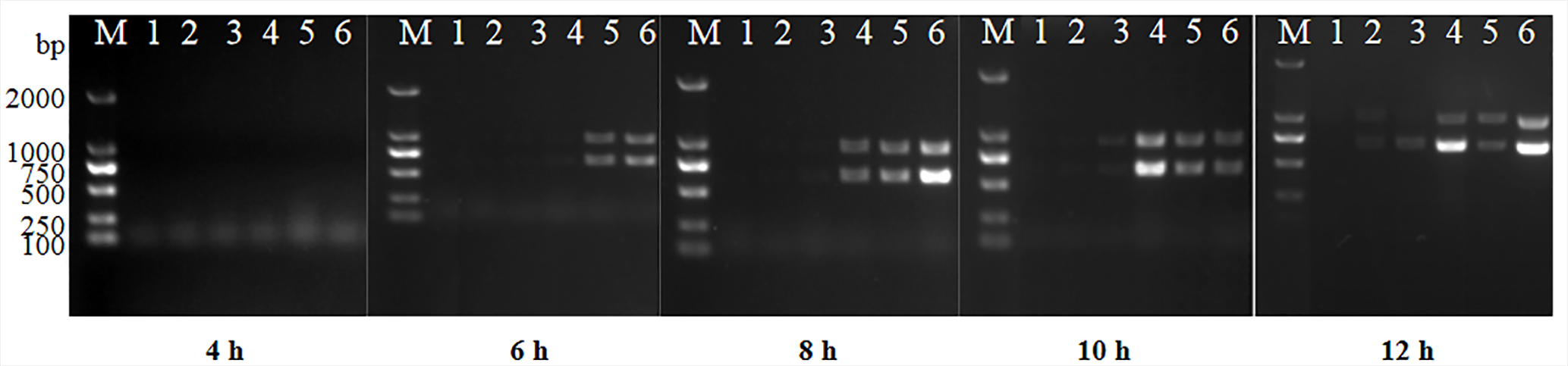

Supplement: FIGURE S1 — Detection results of pork meat contaminated with serotype 4h L. monocytogenes by multiplex PCR. Lanes 1–6: the initial bacterial inoculation dose was 0, 1.8×100, 1.8×101, 1.8×10^2^, 1.8×10^3^, and 1.8×10^4^ CFU, respectively; Lane M: DL2000 DNA ladder. [file Image_1.tif]
